# Allelotype influence at glutathione S-transferase M1 locus on breast cancer susceptibility

**DOI:** 10.1038/sj.bjc.6690055

**Published:** 1999-01

**Authors:** J Charrier, C M Maugard, B Le Mevel, Y J Bignon

**Affiliations:** Unité d'Oncologie Génétique Moléculaire, Centre René Gauducheau,CRLCC Nantes-Atlantique, Bd. J. Monod, Nantes-Saint-Herblain, Cédex 01 44805, France; Laboratoire d'Oncologie Moléculaire, INSERM CRI 9502 et EA 2145, Centre Jean Perrin,58 Rue Montalembert, BP 392, Clermont-Ferrand, Cédex 1, 63011, France

**Keywords:** cancer susceptibility, breast cancer, glutathione S-transferase, allelic polymorphism

## Abstract

The influence of polymorphisms of the glutathione S-transferase gene *GSTM1* in breast cancer susceptibility has been assessed in this study. Previous studies correlated the absence of the GSTM1 protein with an increased risk of developing some cancers, especially lung or bladder cancers, in heavy smokers. In this study, we determined *GSTM1* polymorphisms in a population of 437 female controls from the west of France and 361 community breast cancer patients. Three distinct alleles of this gene exist: GSTM1* A, GSTM1*B and GSTM1*0 (deleted allele). Null subjects (*GSTM1* null) are homozygous for this deletion. The comparative analysis of *GSTM1* allelotypes in our two populations did not demonstrate a statistically significant difference in distribution (*P* = 0.22), although the null genotype was more frequent in cancer patients. However, breast cancer risk was increased in null subjects ≥ 50 years of age compared with non-null subjects [odds ratio = 1.99 (1.19–3.32), *P* = 0.009], but not in null subjects < 50 years of age compared with non-null subjects (*P* = 0.86). Our results suggest that the *GSTM1* null genotype may play a role in post-menopausal breast cancer development. They also point to a putative protective role of the A allele in the older female control group, especially in hemizygous subjects [odds ratio = 0.42 (0.23–0.77), *P* = 0.03]. © 1999 Cancer Research Campaign


*British Journal of Cancer* (1999) **79**(2), 346–353
© 1999 Cancer Research Campaign

### Allelotype influence at glutathione S-transferase M1 locus on breast cancer susceptibility

**J Charrier[1,2]*, CM Maugard[1,2]*, B Le Mevel[1] and YJ Bignon[2]**

[1]Unité d'Oncologie Génétique Moléculaire, Centre René Gauducheau, CRLCC Nantes-Atlantique, Bd. J. Monod, 44805 Nantes-Saint-Herblain Cédex 01, France; [2]Laboratoire d'Oncologie Moléculaire, INSERM CRI 9502 et EA 2145, Centre Jean Perrin, 58 Rue Montalembert, BP 392, 63011 Clermont-Ferrand Cédex 1, France

**Summary** The influence of polymorphisms of the glutathione S-transferase gene *GSTM1* in breast cancer susceptibility has been assessed in this study. Previous studies correlated the absence of the GSTM1 protein with an increased risk of developing some cancers, especially lung or bladder cancers, in heavy smokers. In this study, we determined *GSTM1* polymorphisms in a population of 437 female controls from the west of France and 361 community breast cancer patients. Three distinct alleles of this gene exist: *GSTM1** A, *GSTM1**B and *GSTM1**0 (deleted allele). Null subjects (*GSTM1* null) are homozygous for this deletion. The comparative analysis of *GSTM1* allelotypes in our two populations did not demonstrate a statistically significant difference in distribution ($P = 0.22$), although the null genotype was more frequent in cancer patients. However, breast cancer risk was increased in null subjects ≥ 50 years of age compared with non-null subjects [odds ratio = 1.99 (1.19–3.32), $P = 0.009$], but not in null subjects < 50 years of age compared with non-null subjects ($P = 0.86$). Our results suggest that the *GSTM1* null genotype may play a role in post-menopausal breast cancer development. They also point to a putative protective role of the A allele in the older female control group, especially in hemizygous subjects [odds ratio = 0.42 (0.23–0.77), $P = 0.03$].

**Keywords:** cancer susceptibility; breast cancer; glutathione S-transferase; allelic polymorphism

The incidence of breast cancer has increased in Western countries since the 1980s. Breast cancer represents the most common cause of cancer death in women. Identifying factors that may influence susceptibility to this disease is an important challenge. Several risk factors are well known, such as age at first pregnancy, age at start of puberty, age at menarche, duration of lactation (Franceschi et al, 1990), body mass index (Katoh et al, 1994) and diet (Hunter and Willet, 1994). Epidemiological studies have also shown the role of a family history as a powerful predictor for breast cancer risk (Claus, 1994). Genetic factors are believed to account for at least 5% of all cases of breast cancer (Easton et al, 1993). Rapid developments in molecular genetics have allowed the identification of two major breast cancer susceptibility genes involved in early-onset breast cancer families (Miki et al, 1994; Wooster et al, 1995). However, recent data collected worldwide (Szabo and King, 1997) suggest that germinal mutations of either *BRCA1* or *BRCA2* are observed at a lower rate than expected in breast or breast–ovarian cancer families. Therefore, other susceptibility genes have yet to be identified.

It is well known that up to 80% of human cancers arise as a consequence of environmental exposure (Doll and Peto, 1981). Many compounds in their native or metabolized forms are able to affect DNA integrity and may lead to cancer if exposure persists. Accumulation of these genetic alterations, together with spontaneous DNA replication errors, not corrected by DNA repair systems, could lead to development and/or progression of primary breast cancers.

*Received 18 November 1997*
*Revised 8 May 1998*
*Accepted 13 May 1998*

*Correspondence to:* CM Maugard

*The first two authors contributed equally to this work.

The first line of defence is provided by the ability to metabolize and detoxify xenobiotic toxins (Smith et al, 1995). Inhibition of phase I enzymes, which are involved in carcinogen activation, and/or activation of phase II enzymes, which normally play a detoxifying role, should be able to protect cells against carcinogenic effects of genotoxins. Therefore, deletion or mutation of the genes coding for these phase II enzymes may be responsible for individual or even inherited susceptibility to environmental or endogenous factors, thus predisposing their carriers to the development of cancer in the presence of such genomic insults.

Glutathione S-transferase (GST) genes encode a family of detoxifying phase II enzymes (EC 2.5.1.18), catalysing the conjugation of glutathione to electrophilic compounds. Substrates include exogenous carcinogens and products of oxidative stress. A member of the GST gene superfamily, *GSTM1*, has been mapped to 1p13 (Pearson et al, 1993). Three different alleles of this gene have been identified: *GSTM1** A; *GSTM1** B, which differs from the A allele by a point mutation in exon 7, thus introducing a *Hae*II restriction site in the gene sequence (Fryer et al, 1993); and *GSTM1** 0, which represents the absence of its allele (Seidegard et al, 1988). When this deletion is homozygous, it confers the null genotype on the carrier (Seidegard and Pero, 1988; Seidegard et al, 1988; Strange et al, 1991). The frequency of the null genotype varies from 30% to 80%, depending on the ethnic group studied (Lin et al, 1994). The protein encoded by this gene primarily catalyses the detoxification of alkyl and polycyclic aromatic hydrocarbons (PAHs), intermediate forms of many carcinogens, specifically metabolically generated epoxide intermediates of benzo(a)pyrene. Furthermore, the protein is also able to reduce some superoxides (Smith et al, 1995) and the products of oxidative

Preliminary results of this study were presented as oral communication during the international conference on glutathione and related enzymes in Hilton Head (North Carolina, October 1996).

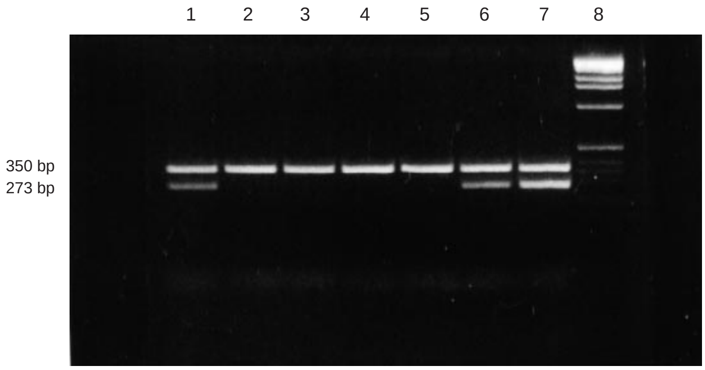

**Figure 1** PCR for identification of *GSTM1* null and non-null genotypes. Agarose gel of PCR products showing the 273-bp DNA fragment amplified from *GSTM1* with a 350-bp sequence from the *PSA* gene as internal control. From left to right: lanes 1 and 6, hemizygote subjects; lanes 2, 3, 4 and 5, *GSTM1* null subjects; lane 7, homo- or heterozygous subject; lane 8, DNA size marker (1-kb ladder). Note that the intensity of the *GSTM1* band amplified from the hemizygotes is inferior to that of the internal *PSA* control band, in contrast to the homo-heterozygote, in whom the intensity is about equal. The *GSTM1* band is absent in the *GSTM1* null subjects, without loss of the PSA band stress, such as DNA hydroperoxides. Previous studies have demonstrated that subjects with the *GSTM1* null genotype or phenotype have an increased risk of developing several cancers, notably lung cancer in heavy smokers (Seidegard et al, 1986; Seidegard et al, 1990; Nazart-Stewart, 1993). However, because of the lack of consensus (Strange et al, 1991), and the existence of ethnic differences in the *GSTM1* genotype distribution (Lin et al, 1994), each type of cancer must be investigated in the context of a specific ethnic background before using the *GSTM1* 0 genotype as a biomarker to define high-risk subjects.

Several studies have investigated the *GSTM1* null genotype prevalence in case–control studies of breast cancer without demonstrating a statistically significant difference between cases and controls. Zhong et al (1993) compared 225 controls and 197 breast cancer patients; Harada et al (1992) studied 84 controls and 65 patients. Ambrosone et al (1995) restricted their study to post-menopausal women: they compared 212 Caucasian patients and 282 community controls and discovered a trend towards an increased breast cancer risk among *GSTM1* null post-menopausal women under 58 years of age [odds ratio (OR) = 2.44]. Paradiso et al (1994) did not detect a difference for the null phenotype frequency among 63 breast cancer patients and 45 healthy patients. In the current study on a broad series of 361 prevalent breast cancers and 437 female controls, we investigated the influence of the *GSTM1* null genotype and of the different *GSTM1* alleles for susceptibility to breast cancer. Allelism at these loci was studied using polymerase chain reaction (PCR) for *GSTM1* null genotype determination and allele-specific PCR for A and B allele identification.

### MATERIALS AND METHODS

### Study population and sample collection

A total of 361 female Caucasian patients from the west of France bearing histologically proven breast cancer (mean age 51 ± 10, range 26–80), were recruited in the René Gauducheau Cancer Centre in Nantes between 1993 and 1996. Control blood samples were obtained from 437 community Caucasian females without any neoplasic disease (mean age 47 ± 13, range 18–101). The protocol had received local ethics committee approval.

Blood (10 ml) was collected in EDTA from all patients. Red cells were lysed after two washes of 1 h each with cold Tris–EDTA buffer (20 mM Tris, 5 mM EDTA). DNA was extracted using a standard phenol–chloroform extraction procedure after proteolysis with proteinase K as described by Sambrook et al. (1989).

### PCR for identification of *GSTM1* null and non-null genotypes

A 273-bp fragment was amplified by multiplex PCR with *GSTM1* primers for exons 4–5 as described by Comstock et al (1990) together with a 350-bp sequence of the prostate-specific antigen gene (*PSA*) as internal control using Moreno's primer sequences (Moreno et al, 1992). DNA templates were amplified in a total volume of 25 µl containing 180 µM of each dNTP, 0.6 µM of each primer, 2.5 mM magnesium chloride, 20 mM Tris-HCl pH 8.55, 16 mM ammonium sulphate, 150 µg $ml^{-1}$ bovine serum albumin (BSA) and 0.65 units of Biotaq DNA polymerase (Bioprobe, Paris, France). After initial denaturation at 94°C (5 min), 25 cycles of amplification, 56°C (30 s), 72°C (1 min) and 94°C (1 min) were performed followed by one cycle at 56°C (30 s) and 72°C (7 min). PCR products were run on 3% agarose gels stained with ethidium bromide. Determination of the homo- or hemizygote presence of the gene was achieved by comparing the intensities of the PCR products of the *GSTM1* gene with those of the *PSA* gene (Figure 1). Mostly, it was possible to show the presence of zero, one or two alleles by direct analysis on the gel, as four samples were systematically introduced as positive controls in each PCR, two *GSTM1* hemizygotes, *GSTM1** A/*GSTM1** 0 and *GSTM1** B/*GSTM1** 0, and two homozygotes, *GSTM1** A/*GSTM1** A and *GSTM1** B/*GSTM1** B. Each DNA template was amplified in at least two different PCRs.

The ratio between the *GSTM1* and *PSA* band intensities was computed by densitometric measurement on the Polaroid negative photograph of the gel. A ratio of 0.3–0.7 was considered to correspond to *GSTM1* hemizygosity and 0.7–1.2 to *GSTM1* homozygosity or heterozygosity (Figure 2).

Linearity of the internal standard amplification with DNA concentrations has been tested using quantities ranging from 10 to 2000 ng. A hemizygous DNA sample and a sample containing two alleles were amplified. The reproducibility of the results was investigated with a hemizygous sample and a sample containing two alleles. Each of them were amplified in the same PCR and in different PCRs.

Validation of the results was performed on 45 non-null samples, randomly selected, each amplified in at least two separate PCRs.

© Cancer Research Campaign 1999

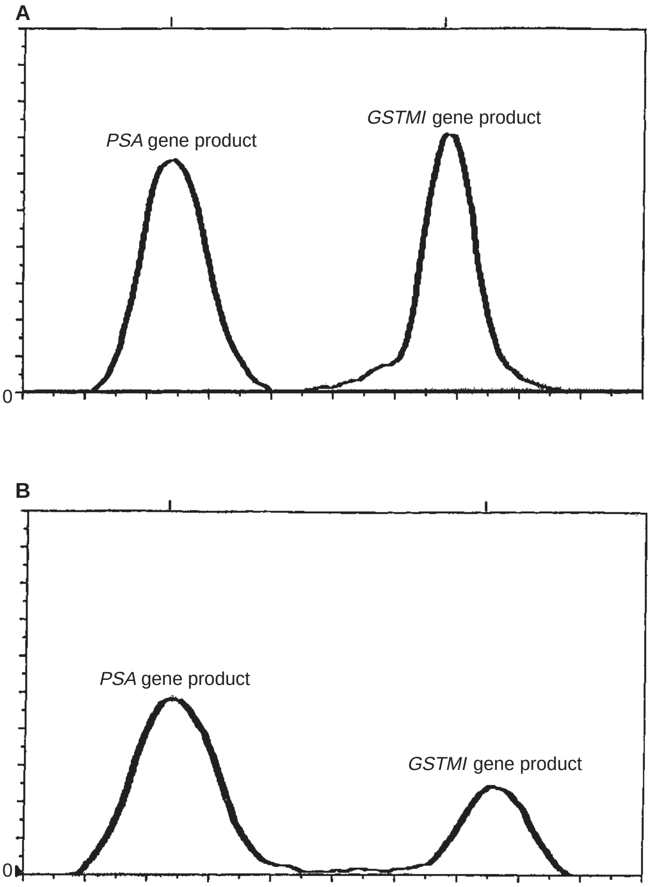

**Figure 2** Quantification of *GSTM1* homo- or hemizygosity. The ratio between the *GSTM1* and *PSA* band intensities was quantified by densitometric measurement of the Polaroid photograph of the gel. A ratio of 0.3–0.7 was considered to correspond to hemizygosity (**A**) and 0.8–1.2 to homozygosity (**B**)

Finally, the results described in this paper were obtained using 100 ng of DNA template for each sample.

### PCR for identification of *GSTM1* alleles A and B

Identification of one of the *GSTM1* genotypes – *GSTM1** A / *GSTM1** B, *GSTM1** A / *GSTM1** A, *GSTM1** B / *GSTM1** B, *GSTM1** A / *GSTM1** 0 or *GSTM1**B / *GSTM1**0 – was achieved by PCR amplification of the homozygotes, heterozygotes and hemizygotes for *GSTM1* DNA using the method previously described by Fryer et al (1993*a*). An allele-specific PCR was performed as follows: 300 ng of genomic DNA was amplified in a volume of 50 µl containing 1.5 mM magnesium chloride, 200 µM of each dNTP, 600 nM of each primer, 1.5 units of Amplitaq (Perkin Elmer Cetus, Paris, France) and Amplitaq buffer. DNA from patients positive for *GSTM1* alleles resulted in a 132-bp fragment. β-Globin primers (600 nM) were added as control for amplification and resulted in the production of a 299-bp DNA fragment.

After initial denaturation at 94°C (5 min), 30 cycles of 94°C (45 s), 57°C (1 min) and 72°C (1.5 min) were performed. Confirmation of the presence of the A or B allele was accomplished by restriction of 20 µl of the PCR product with *Hae*II (4 h at 37°C). Migration on 4% agarose gels allowed the distinction between the non-digested B allele (132 bp) and the digested A allele (112 bp).

The accuracy of our methods of identifying allele number and distinguishing hemizygosity for A or B alleles was checked by studying the transmission of those alleles among a set of five cancer families, recruited with informed consent during genetic counselling.

### Statistical analysis

Odds ratios (ORs) and 95% confidence intervals (CIs) were calculated for the association of the *GSTM1* null genotype with breast cancer using the Miettinen (1976) calculation method; the hypothesized high-risk category (i.e. *GSTM1* deleted) served as reference category for the model.

Statistical significance was assessed by using the $\chi^2$ test. The observed distribution within breast cancer patients was compared with the observed distribution within the controls according to four different criteria: null and non-null genotype, number of alleles (0, 1 or 2), distribution of the A, 0, B and AB allelotypes, and distribution of the six different genotypes: *GSTM1** A / *GSTM1** 0, *GSTM1** A / *GSTM1** A, *GSTM1** B / *GSTM1** 0, *GSTM1** B / *GSTM1** B, *GSTM1** A / *GSTM1** B and *GSTM1** 0 / *GSTM1** 0.

Unstratified comparison was performed, as well as comparison within strata of age at diagnosis for breast cancer patients or age at sampling for controls. Two groups of subjects were defined: under 50 years of age (< 50 age group) and 50 years of age and over (≥ 50 age group). When one of the calculated values was less than 5 on

 *© Cancer Research Campaign 1999*

**Table 1** Distribution of null and non-null genotypes and of allele numbers in subjects aged under 50 and in subjects aged 50 years old and over

| Subgroups | Patients *n* (%) | Controls *n* (%) | OR | 95% CI | $\chi^2$ | d.f.[c] | *P*-value |
|---|---|---|---|---|---|---|---|
| *Whole group* | 361 | 437 | | | | | |
| *GSTM1* | | | | | | | |
| Null (no alleles) | 201 (55.7) | 224 (51.3) | | | 1.37 | d.f. = 1 | 0.22 NS[a] |
| Non-null | 160 (44.3) | 213 (48.7) | 0.84 | (0.63–1.11) | | | |
| *GSTM1* | | | | | | | |
| Null (no alleles) | 201 (55.7) | 224 (51.3) | | | 1.71 | d.f. = 2 | 0.43 NS[a] |
| One allele | 139 (38.5) | 182 (41.6) | 0.85 | (0.64–1.14) | | | |
| Two alleles | 21 (5.8) | 31 (7.1) | 0.75 | (0.42–1.35) | | | |
| *< 50 age group* | 226 | 330 | | | | | |
| *GSTM1* | | | | | | | |
| Null (no alleles) | 120 (53.1) | 178 (53.9) | | | 0.012 | d.f. = 1 | 0.86 NS |
| Non-null | 106 (46.9) | 152 (46.1) | 1.03 | (0.74–1.45) | | | |
| *GSTM1* | | | | | | | |
| Null (no alleles) | 120 (53.1) | 178 (53.9) | | | 1.81 | d.f. = 2 | 0.40 NS |
| One allele | 93 (41.1) | 124 (37.6) | 1.11 | (0.78–1.59) | | | |
| Two alleles | 13 (5.8) | 28 (8.5) | 0.69 | (0.34–1.38) | | | |
| *≥ 50 age group* | 135 | 107 | | | | | |
| *GSTM1* | | | | | | | |
| Null (no alleles) | 81 (60.0) | 46 (43.0) | 1.99[d] | (1.19–3.32)[e] | 6.26 | d.f. = 1 | 0.009 S*[b] |
| Non-null | 54 (40.0) | 61 (57.0) | 0.50 | (0.30–0.84) | | | |
| *GSTM1* | | | | | | | |
| Null (no alleles) | 81 (60.0) | 46 (43.0) | | | 10.27 | d.f. = 2 | 0.006 S* |
| One allele | 46 (34.1) | 58 (54.2) | 0.45 | (0.27–0.76) | 8.06 | d.f. = 1 | 0.003 S* |
| Two alleles | 8 (5.9) | 3 (2.8) | 1.51 | (0.39–5.94) | | | not available |

*GSTM1* 0 and *GSTM1* non-null genotype frequencies in the whole population and within the subgroups of patients and controls were compared according to age. No significant difference was observed between patients and controls in the whole population; the null genotype was significantly predominant in the group of patients aged 50 years old and over compared with the same population of controls. Comparison between the number of *GSTM1* alleles in patients and controls in the whole population and within the different subgroups of age is detailed. No significant difference was observed between patients and controls in the whole population for breast cancer risk; hemizygotes were predominant in the control population aged 50 and over. [a]NS, non-significant; [b]S* statistically significant; [c]df, degrees of freedom; [d]OR calculated with the lowest risk *GSTM1* non-null as reference; [e]confidence interval.

the contingency tables, the Fisher exact test was used. Statistical analyses were performed using the Statview package, version 4.02.

### RESULTS

### PCR linearity

Linearity of PCR co-amplification of *GSTM1* and *PSA* product was checked over a 10–2000 ng range of DNA template quantities.

For the hemizygote sample, a good linearity was obtained between 10 and 300 ng with a correlation coefficient $R^2 = 0.86$. When the sample contained two alleles, very good linearity was observed between 10 and 300 ng with a correlation coefficient $R^2 = 0.98$ (data not shown).

### PCR reproducibility

Good inter- and intra-assay reproducibility for allele number determination was observed for samples amplified ten times in the same PCR and in ten different PCRs for a one-allele (i.e. hemizygous) sample; the computed ratios between GSTM1 band and PSA band intensities were, respectively, 0.62 ± 0.05 and 0.63 ± 0.06. A sample containing two alleles was amplified seven times in the same PCR and in nine different PCRs, resulting ratios were respectively 0.99 ± 0.07 and 0.98 ± 0.09 (data not shown).

### Quantitative PCR results

Among the non-null samples, 45 were selected at random to undergo densitometric measurement. PCRs used for quantification on these samples were repeated at least twice (mean number of PCRs 2.8 ± 1.5, range 2–9). By direct comparison, on the electrophoresis gel, 26 subjects were determined as carrying one allele, so they were called hemizygous for *GSTM1*. Nineteen subjects had two alleles. In those cases, further identification with a mutated allele-specific amplification (MASA) PCR was needed to distinguish between the *GSTM1** A and the *GSTM1** B alleles and to conclude if they were homo- or heterozygous for those alleles.

Densitometric measurement of GSTM1 band intensity compared with PSA band intensity gave an average ratio of absorbance equal to 0.59 ± 0.07 for the one-allele samples (range 0.40–0.65) and 0.94 ± 0.09 for the two-allele samples (range 0.80–1.18).

These results were in complete accordance with results obtained on the same samples by direct comparison on the gel.

### Population mean age in null and non-null genotypes

No statistically significant difference in mean age according to null and non-null status was found within the control subjects or breast cancer patients in our study. In the control group, mean age

© Cancer Research Campaign 1999

**Table 2** Distribution of different *GSTM1* genotypes in subjects aged under 50 and in subjects aged 50 years and over

| Subgroups of population N | Patients n (%) | Controls n (%) | OR | 95% CI | χ² | d.f.[c] | P-value |
|---|---|---|---|---|---|---|---|
| *Whole group* | 361 | 437 | | | | | |
| Null | 201 (55.7) | 224 (51.3) | | | 2.96 | d.f. = 3 | 0.39 NS |
| A | 87 (24.1) | 128 (29.3) | 0.76 | (0.54–1.06) | | | |
| B | 59 (16.3) | 71 (16.2) | 0.93 | (0.62–1.37) | | | |
| A/B | 14 (3.9) | 14 (3.2) | 1.11 | (0.52–2.39) | | | |
| Null | 201 (55.7) | 224 (51.3) | | | 4.39 | d.f. = 5 | 0.49 NS[a] |
| | 82 (22.7) | 117 (26.8) | 0.78 | (0.56–1.10) | | | |
| | 5 (1.4) | 11 (2.5) | 0.91 | (0.18–1.46) | | | |
| | 57 (15.8) | 66 (15.1) | 0.96 | (0.64–1.44) | | | |
| | 2 (0.5) | 5 (1.1) | not available | | | | |
| A/B | 14 (3.9) | 14 (3.2) | 1.11 | (0.52–2.39) | | | |
| *< 50 age group* | 226 | 330 | | | | | |
| Null | 120 (53.1) | 178 (54.0) | | | 0.76 | d.f. = 3 | 0.85 NS |
| A | 57 (25.2) | 89 (27.0) | 0.95 | (0.63–1.42) | | | |
| B | 41 (18.1) | 51 (15.4) | 1.19 | (0.74–1.91) | | | |
| A/B | 8 (3.5) | 12 (3.6) | 0.99 | (0.39–2.49) | | | |
| Null | 120 (53.1) | 178 (54.0) | | | 3.89 | d.f. = 5 | 0.56 NS |
| A/0 | 53 (23.5) | 78 (23.6) | 1.01 | (0.66–1.53) | | | |
| A/A | 4 (1.8) | 11 (3.3) | 0.54 | (0.17–1.71) | | | |
| B/0 | 40 (17.7) | 46 (13.9) | 1.29 | (0.81–2.09) | | | |
| B/B | 1 (0.4) | 5 (1.5) | not available | | | | |
| A/B | 8 (3.5) | 12 (3.6) | 0.99 | (0.39–2.49) | | | |
| *≥ 50 age group* | 135 | 107 | | | | | |
| Null | 81 (60.0) | 46 (43.0) | | | 9.81 | d.f. = 3 | 0.02 S*[b] |
| A | 30 (22.2) | 39 (36.4) | 0.44 | (0.24–0.79) | | | |
| B | 18 (13.3) | 20 (18.7) | 0.51 | (0.25–1.06) | | | |
| A/B | 6 (4.4) | 2 (1.9) | not available | | | | |
| Null | 81 (60.0) | 46 (43.0) | | | 12.28 | d.f. = 5 | 0.03 S* |
| A/0 | 29 (21.5) | 39 (36.4) | 0.42 | (0.23–0.77) | | | |
| A/A | 1 (0.7) | 0 (0.0) | not available | | | | |
| B/0 | 17 (12.6) | 20 (18.7) | 0.48 | (0.23–1.01) | | | |
| B/B | 1 (0.7) | 0 (0.0) | not available | | | | |
| A/B | 6 (4.4) | 2 (1.9) | 1.70 | (0.34–8.64) | | | |

Comparison between *GSTM1* alleles A, B, AB and 0 in patients and controls in the whole population and in the different subgroups according to age. No significant difference was observed between patients and controls in the whole population; the null genotype and the AB genotype were predominant in the group of patients age 50 and over compared with the same population of controls. In contrast, the A allele was predominant in the control population aged 50 and over. The A/0 genotype was predominant in the control population of 50 years old and over. [a]NS, non-significant; [b]S*, statistically significant; [c]d.f., degrees of freedom.

was 65.1 years in null and 65.2 years in non-null subjects ≥ 50 years of age ($P$ = 0.50) and 42.1 years in null and 41.5 years in non-null subjects <50 years of age ($P$ = 0.94).

In our breast cancer population, mean age was 61.9 years in null and 63.3 years in non-null patients ≥50 years of age ($P$ = 0.67) and 43.9 years in null and 45.3 years in non-null patients <50 years of age ($P$ = 0.26).

### Distribution of null and non-null genotypes

Of the 437 female controls, 51.3% had the *GSTM1* null genotype, 7.1% had two alleles and 41.6% were hemizygous within this group (Table 1). The null genotype was more frequent in the < 50 age subgroup (53.9%) than in the ≥ 50 age subgroup, in which the observed frequency was only 43%. This 10.9% difference (53.9% vs 43%) was statistically significant ($P$ = 0.05).

Of 361 breast cancer patients, 55.7% were *GSTM1* null, 5.8% had two alleles and 38.5% were hemizygous (Table 1). In contrast to the control group, the null genotype was less frequent in the < 50 age subgroup (53.1%) than in the ≥ 50 age subgroup, in which its frequency reached 60%. However, the 6.9% observed (60% vs 53.1%) difference was not statistically significant ($P$ = 0.23).

There was no significant difference when we compared the whole affected and control groups or the < 50 age groups for null and non-null genotype distribution. However, a statistically significant difference was observed in the ≥ 50 age subgroup (OR = 1.99; 95% CI 1.19–3.32; $P$ = 0.009; Table 1) with a clear predominance of the null genotype in the patient group (60%) compared with the control group (43%) (Table 1).

### Distribution of *GSTM1* allele number

Within the total population, most of the subjects had either no or one allele of *GSTM1* (94.2% of the subjects < 50 years of age and 94.1% of the subjects ≥ 50 years of age). In this total population, as well as in the < 50 age group, the number of alleles was not significantly different between patients and controls ($P$ = 0.43 and $P$ = 0.86 respectively).

 © Cancer Research Campaign 1999

However, in the ≥ 50 age group, people with one allele were significantly more common in the control group than in the patient group ($P$ = 0.006) (Table 1). Moreover, in this subgroup, cancer risk related to the presence of one allele was significantly decreased in hemizygous subjects compared with null subjects (OR = 0.45; 95% CI 0.27–0.76; $P$ = 0.003) (Table 2). This was not observed when we compared subjects who had two alleles with null subjects. The significance of this last comparison was not calculated because of the small size of the two allele groups (Table 2).

Thus, the risk of breast cancer occurrence was reduced in hemizygous subjects ≥ 50 years of age. However, the role of the presence of two alleles in determination of the breast cancer risk could not be examined owing to the small size of this subgroup.

### Distribution of A and B allelotypes in non-null subjects

Among non-null subjects, the A allelotypes were more frequent, B allelotypes were less frequent and A/B was rare without any statistically significant difference in distribution between cases and controls (Table 2). Surprisingly, in the ≥ 50 age control group, the A allelotypes were more prominent than in the ≥ 50 age patient group; the B allelotypes were slightly over-represented in the ≥ 50 age control group and A/B carriers were less frequent. This difference in allelotype distribution was statistically significant ($P$ = 0.02) (Table 2). Moreover, the A/0 and B/0 genotypes were over-represented in the ≥ 50 age control group when compared with the ≥ 50 age patient group, with a statistically significant difference ($P$ = 0.03). The A/0 genotype and, to a lesser extent, the B/0 genotype were responsible for a decreased risk of breast cancer in the ≥ 50 age group (OR = 0.42, 95% CI, 0.23–0.77, and OR = 0.48, 95% CI 0.23–1.01, respectively) (Table 2).

### DISCUSSION

Although the *GSTM1* null genotype in our study was more frequent in breast cancer cases than in controls, this difference does not reach a statistical significance in the overall population. This is consistent with results previously reported for smaller groups by Harada et al (1992), Zhong et al (1993) or Paradiso et al (1994) comparing null and non-null genotypes.

As we cannot take into account the precise hormonal status of women in our series, because these data were not recorded in most of our controls and were missing for some of the cancer patients, we examined the influence of allelism at the *GSTM1* locus on breast cancer susceptibility according to subsequent subcategories: (1) women < 50 years of age and (2) women ≥ 50 years of age. This subdivision best approximates the division according to the menopausal status.

Within individuals aged 50 years old and over, we identified a statistically significant increase in null genotype frequency in cancer patients ($P$ = 0.009, OR = 1.98). Ambrosone et al (1995) did not find any increased breast cancer risk linked to the *GSTM1* null genotype. However, they detected a trend to an elevated, but not statistically significantly increased, risk when they studied young post-menopausal patients (under 58 years old). The discrepancy between their results and ours in women 50 years old and over may be due to a difference in recruited population characteristics: in their study, cases with the null genotype were significantly younger than those with the non-null genotype; this was not the case in our series. Taken together, these results suggested a putative role of *GSTM1* in breast cancer susceptibility in the post-menopausal group. Our data do suggest a role for the null genotype in late-onset breast cancer susceptibility with an OR of 1.99 (95% CI 1.19–3.32).

In the < 50 years of age breast cancer-affected population, other genes, such as the two major predisposition genes, *BRCA1* and *BRCA2* are known to play the most important role in breast cancer susceptibility (Miki et al, 1995; Wooster et al, 1995; Rebbeck et al, 1996). However, those genes are not sufficient to explain breast cancer susceptibility in every cancer-prone family (Easton et al, 1997). Our current data, as well as previous results, do not suggest any role of *GSTM1* in the early onset of breast cancer.

The null genotype was 10.9% less frequent in the ≥ 50 age controls than in the < 50 age control subgroups and 6.9% more frequent in the ≥ 50 age patients than in the patients in the < 50 age subgroup (Table 1). These data suggest that some of the *GSTM1* null subjects who did not develop breast cancer may have died from neoplastic diseases other than breast cancer, or from various other pathologies. The role of the null genotype in diseases such as inflammatory diseases has already been reported, for example in patients suffering from Crohn's disease (Duncan et al, 1995) or systemic lupus erythematosus with a $Ro^+/La^-$ autoantibody profile (Ollier et al, 1996).

We also studied non-null subjects for the presence of either A or B alleles of the *GSTM1* gene and distinguished between homozygotes A/A, B/B, heterozygotes A/B and hemizygotes A/0 and B/0. This allowed us to investigate the putative protective role of these particular alleles, as well as a dosage effect of these alleles.

In the ≥ 50 age group, the A allelotype was more prominent in the control group than in the patient population (Table 2); a slightly higher incidence of the B allelotype was also observed in the control group. Moreover, a higher incidence of A/0 and B/0 genotypes was found in the control group (Table 2). The corresponding OR suggested a protective effect of a single allele in these subsets of the population. Interestingly, this protective effect was not observed with the A/B genotype. However, this subgroup is too small to yield meaningful statistics. It will be of interest to investigate the differential role of the A/A vs the A/0 genotype, and of the B/B vs the B/0 genotype, in order to determine if a different dosage of these alleles is playing a role in breast cancer susceptibility. However, the number of subjects in our study was not sufficient to identify statistically significant differences, and the current study will have to be extended to investigate this hypothesis.

Distinguishing between the A and B alleles seems to be interesting because their role may be different: Fryer et al (1993*b*) demonstrated that the B allele confers better protection against pituitary adenomas than does the A allele, and Duncan et al (1995) also observed a putative protective effect of the B allele in Crohn's disease. Heagerty et al (1994) and Yengi et al (1996) have shown a protective effect of the *GSTM1**A and *GSTM1** B alleles in cutaneous basal cell carcinoma but did not distinguish (A/A) and (B/B) from respectively (A/0) and (B/0), so the influence of the allele number cannot be assessed by their study.

Like previous studies, our data failed to find a statistically significant prevalence of the *GSTM1* null genotype in breast cancer when we analysed the complete population. The effect of the null genotype could be insufficient by itself to favour cancer development. Studying other susceptibility genes could be helpful to define a 'high-risk haplotype'.

The effect of interaction of *GSTM1* with other polymorphic genes encoding detoxifying enzymes is of interest. For example,

© Cancer Research Campaign 1999

the *Cyp1A1* allele was found to increase the risk of lung cancer in Japanese *GSTM1* null subjects (Nakachi et al, 1993).

We cannot exclude the existence of interactions between *GSTM1* and major predisposition genes. Such interactions have already been demonstrated for other genes such as *HRAS1*. Phelan et al (1996) showed that carriers of *BRCA1* mutations, harbouring one or two rare alleles of *HRAS1*, had an ovarian cancer risk that was 2.11-fold higher than that of those carrying common alleles of *HRAS1*.

Environmental factors or diet intake may increase the occurrence of a malignant phenotype in *GSTM1* null subjects. This was observed for lung cancer where frequent smoking (Kihara et al, 1994) or low vitamin C consumption (Garcia-Closas et al, 1995) results in a higher cancer risk in the *GSTM1* 0 population than in the non-null population.

In conclusion, we hypothesize, based on our current data, that long-term exposure to environmental or endogenous carcinogenic factors could favour breast cancer development in the ≥ 50 age group in previously healthy patients bearing the null genotype. Moreover, the null genotype could predispose to other pathologies. That is why we propose that the null genotype may be responsible for a lower life expectancy. The follow-up, in a longitudinal study, of our control group, to register each pathology developed by null and non-null subjects will allow us to investigate this hypothesis.

Integration of germline DNA genotyping for polymorphisms of different carcinogen-metabolizing enzymes and information about dietary habits, lifestyle and family history in a prospective case–control study will be necessary to elucidate further the role of interaction between different genes for carcinogen-metabolizing enzymes and between these genes and environmental factors.

### ABBREVIATIONS

GST, glutathione S-transferase; PCR, polymerase chain reaction; MASA, mutated allele specific amplification; PAH, polyaromatic hydrocarbon; OR, odds-ratio; CI, confidence interval

### ACKNOWLEDGEMENTS

The authors wish to thank F Noël for her help in registering patients and controls, P Perrin and M Denis for technical assistance, L Campion and F Kwiakowsky for their help with statistical analysis, JY Muller and M Hamidou for providing control blood samples, all the clinicians of Centre René Gauducheau for breast cancer patients recruitment, F Ali-Osman and M Kuiper for helpful comments and discussion on this manuscript. This work was supported by La Ligue Nationale contre le Cancer, les Comités départementaux de Loire-Atlantique et de Vendée de La Ligue Nationale, les Lyon's club de la Baule et de Nantes Val d'Erdre.

### REFERENCES

Ambrosone CB, Freudenheim JL, Graham S, Marshall JR, Vena JE, Brasure JR, Laughlin R, Nemoto T, Michalek AM, Harrington A, Ford TD and Shields PG (1995) Cytochrome *P450 1A1* and glutathione S-transferase (M1) genetic polymorphisms and postmenopausal breast cancer risk. *Cancer Res* **55**: 3483–3485

Claus EB (1994) Genetic epidemiology of breast cancer in younger women. *J Natl Cancer Inst Monogr* **16**: 49–53

Comstock KE, Sanderson BJS, Claflin G and Henner WD (1990) *GST1* gene deletion determined by polymerase chain reaction. *Nucleic Acids Res* **18**: 3670

Doll R and Peto R (1981) The causes of cancer: quantitative estimates of avoidable risks of cancer in the United States today. *J Natl Cancer Inst* **66**: 1191–1308

Duncan H, Swan C, Green J, Jones P, Brannigan K, Alldersea J, Fryer AA and Strange RC (1995) Susceptibility to ulcerative colitis and Crohn's disease: interactions between glutathione S-transferase *GSTM1* and *GSTT1* genotypes. *Clin Chim Acta* **240**: 53–61

Easton D, Ford D and Peto J (1993) Inherited susceptibility to breast cancer. *Cancer Surv* **18**: 95–113

Easton D (1997) Breast cancer genes – what are the real risks? *Nature Genet* **16**: 210–211

Franceschi S, La Vecchia C, Negri E, Parazzini F and Boyle P (1990) Breast cancer risk and history of selected medical conditions linked with female hormones. *Eur J Cancer* **26**: 781–785

Fryer AA, Zhao L, Alldersea J, Pearson WR and Strange RC (1993a) Use of site-directed mutagenesis of allele-specific PCR primers to identify the *GSTM1* A, *GSTM1* B, *GSTM1* A, B and *GSTM1* null polymorphisms at the glutathione S-transferase, *GSTM1* locus. *Biochem J* **295**: 313–315

Fryer AA, Zhao L, Alldersea J, Boggild MD, Perrett CW, Clayton RN, Jones PW and Strange RC (1993b) The glutathione S-transferases: polymerase chain reaction studies on the frequency of the *GSTM1* 0 genotype in patients with pituitary adenomas. *Carcinogenesis* **14**: 563–566

Garcia-Closas M, Kelsey KT, Wiencke JK and Christiani DC (1995) Nutrient intake as a modifier of the association between smoking-induced lung cancer and glutathione S-tranferase μ deletion. *Proc Am Ass Cancer Res* **36**: 281

Harada S, Misawa S, Nakamura T, Tanaka N, Ueno E and Nozoe M (1992) Detection of *GST1* gene deletion by the polymerase chain reaction and its possible correlation with stomach cancer in Japanese. *Hum Genet* **90**: 62–64.

Heagerty AHM, Fitzgerald D, Smith A, Bowers B, Jones P, Fryer AA, Zhao L, Alldersea J and Strange RC (1994) Glutathione S-transferase GSTM1 phenotypes and protection against cutaneous tumours. *Lancet* **343**: 266–268

Hunter DJ and Willet WC (1994) Diet, body build, and breast cancer. *Annu Rev Nutr* **14**: 393–418

Katoh A, Watzlaf VJM and D'Amico F (1994) An examination of obesity and breast cancer survival in post-menopausal women. *Br J Cancer* **70**: 928–933

Kihara M, Kihara M and Noda K (1994) Lung cancer risk of *GSTM1* null genotype is dependent on the extent of tobacco smoke exposure. *Carcinogenesis* **15**: 415–418

Lin HJ, Han C, Bernstein DA, Hsiao W, Lin BK and Hardy S (1994) Ethnic distribution of the glutathione transferase Mu 1–1 (*GSTM1*) null genotype in 1473 individuals and application to bladder cancer susceptibility. *Carcinogenesis* **15**: 1077–1081

Miettinen O (1976) Estimability and estimation in case-referent studies. *Am J Epidemiol* **103**: 226–235

Miki Y, Swensen J, Shattuck-Eidens D, Futreal PA, Harshman K, Tavtigian S, Liu Q, Cochran C, Bennett LM, Ding W, Bell R, Rosenthal J, Hussey C, Tran T, Mc Clure M, Frye C, Hattier T, Phelps R, Haugen-Strano A, Katcher H, Yakumo K, Gholami Z, Shaffer D, Stone S, Bayer S, Wray C, Bogden R, Dayananth P, Ward J, Tonin P, Narod S, Bristow PK, Norris FH, Helvering L, Morrisson P, Rosteck P, Lai M, Barett JC, Lewis C, Neuhausen S, Cannon-Albright L, Goldgar D, Wiseman R, Kamb A and Skolnick MH (1994) A strong candidate for the breast and ovarian cancer susceptibility gene *BRCA1*. *Science* **266**: 66–71

Moreno JG, Croce CM, Fischer R, Monne M, Vihko P, Mulholland SG and Gomella LG (1992) Detection of hematogenous micrometastasis in patients with prostate cancer. *Cancer Res* **52**: 6110–6112

Nakachi K, Imai K, Hayashi S and Kawajari K (1993) Polymorphisms of the *Cyp1A1* and glutathione S-transferase genes associated with susceptibility to lung cancer in relation to cigarette dose in a Japanese population. *Cancer Res* **53**: 2994–2999

Nazar-Stewart V, Motulsky AG, Eaton DL, White E, Hornung SK, Leng ZT, Stapleton P and Weiss NS (1993) The glutathione S-transferase μ polymorphism as a marker for susceptibility to lung carcinoma. *Cancer Res* **53**: 2313–2318

Ollier W, Davies E, Snowden N, Alldersea J, Fryer A, Jones P and Strange R (1996) Association of homozygosity for glutathione-S-transferase *GSTM1* null alleles with the RO+/La– autoantibody profile in patients with systemic lupus erythematosus. *Arthr Rheum* **39**: 1763–1764

Paradiso A, Vetrugno MG, Capuano G, Longo S, Sibilano L, Schittuli F, Correale M, Barletta A, Pelagio G and De Lena (1994) Expression of GST-μ transferase in breast cancer patients and healthy controls. *Int J Biol Markers* **9**: 219–223

Pearson WR, Vorachek WR, Xu S, Berger R, Hart I, Vannais D and Patterson D (1993) Identification of class-mu glutathione transferase genes *GSTM1–GSTM5* on human chromosome 1p13. *Am J Hum Genet* **53**: 220–233

Phelan CM, Rebbeck TR, Weber BL, Devilee P, Ruttledge MH, Lynch HT, Lenoir GM, Stratton MR, Easton DF, Ponder BA, Cannon-Albright L, Larsson C,

 © Cancer Research Campaign 1999

Goldgar DE and Narod SA (1996) Ovarian cancer risk in *BRCA1* carriers is modified by the *HRAS1* variable number of tandem repeat (VNTR) locus. *Nat Genet* **12**: 309–311

Rebbeck TR, Couch FJ, Kant J, Calzone K, DeShano M, Peng Y, Chen K, Garber JE and Weber BL (1996) Genetic heterogeneity in hereditary breast cancer: role of *BRCA1* and *BRCA2*. *Am J Hum Genet* **59**: 547–553

Sambrook J, Fritsch EF and Maniatis T (1989) Commonly used techniques in molecular cloning. Purification of nucleic acids. Extraction with phenol: chloroform. In *Molecular Cloning*. *A laboratory manual*, 2nd edn, pp. E3–E4 Sambrook J, Fritsch EF, Maniatis T (eds) Cold Spring Harbor Laboratory Press: Cold Spring Harbor, NY

Seidegard J, Pero RW, Miller DG and Beattie EJ (1986) A glutathione transferase in human leucocytes as a marker for the susceptibility to lung cancer. *Carcinogenesis* **7**: 751–753

Seidegard J and Pero RW (1988) The genetic variation and the expression of human glutathione transferase mu. *Klin Wochenschr* **66**: 125–126

Seidegard J, Vorachek WR, Pero RW and Pearson WR (1988) Hereditary differences in the expression of the human glutathione transferase active on *trans*-stilbene oxide are due to a gene deletion. *Proc Natl Acad Sci USA* **85**: 7293–7297

Seidegard J, Pero RW, Markowitz MM, Roush G, Miller DG and Beattie EJ (1990) Isoenzyme(s) of glutathione transferase (class Mu) as a marker for the susceptibility to lung cancer: a follow-up study. *Carcinogenesis* **11**: 33–36

Smith G, Stanley LA, Sim E, Strange RC and Wolf CR (1995) Metabolic polymorphisms and cancer susceptibility. *Cancer Surv* **25**: 27–65

Strange RC, Matharoo B, Faulder GC, Jones P, Cotton W, Elder JB and Deakin M (1991) The human glutathione S-transferases: a case–control study of the incidence of the GST1 0 phenotype in patients with adenocarcinoma. *Carcinogenesis* **12**: 25–28

Szabo CI and King MC (1997) Population genetics of *BRCA1* and *2*. *Am J Hum Genet* **60**: 1013–1020

Wooster R, Bignell G, Lancaster J, Swift S, Seal S, Mangion J, Collins N, Gregory S, Gumbs C, Micklem G, Barfoot R, Hamoudi R, Patel S, Rice C, Biggs P, Hashim Y, Smith A, Connor F, Arason A, Gudmundsonn J, Ficenec D, Kelsell D, Ford D, Tonin P, Bishop DT, Spurr NK, Ponder BAJ, Eeles R, Peto J, Devilee P, Cornelisse C, Lynch H, Narod S, Lenoir G, Egilsson V, Barkadottir RB, Easton DF, Bentley DR, Futreal PA, Ashworth A and Stratton MR (1995) Identification of the breast cancer susceptibility gene *BRCA2*. *Nature* **378**: 789–792

Yengi L, Inskip J, Gilford J, Alldersea J, Bailey L, Lear JT, Heagerty AH, Bowers P, Hand P, Hayes JD, Jones PW, Strange RC and Fryer AA (1996) Polymorphism at the glutathione S-transferase locus *GSTM3*: interactions with cytochrome P450 and glutathione S-transferase genotypes as risk factors for multiple cutaneous basal cell carcinoma. *Cancer Res* **56**: 1974–1977

Zhong S, Wyllie AH, Barnes D, Wolf CR and Spurr NK (1993) Relationship between the *GSTM1* genetic polymorphism and susceptibility to bladder, breast and colon cancer. *Carcinogenesis* **14**: 1821–1824

© Cancer Research Campaign 1999
